# Sphingolipids as prognostic biomarkers of neurodegeneration, neuroinflammation, and psychiatric diseases and their emerging role in lipidomic investigation methods

**DOI:** 10.1016/j.addr.2020.04.009

**Published:** 2020-04-28

**Authors:** Daan van Kruining, Qian Luo, Gerhild van Echten-Deckert, Michelle M. Mielke, Andrew Bowman, Shane Ellis, Tiago Gil Oliveira, Pilar Martinez-Martinez

**Affiliations:** a Division of Neuroscience, School for Mental Health and Neuroscience, Faculty of Health, Medicine, and Life Sciences, Maastricht University, Maastricht, the Netherlands; b LIMES Institute for Membrane Biology and Lipid Biochemistry, Kekulé-Institute, University of Bonn, Bonn, Germany; c Department of Health Sciences Research and Department of Neurology, Mayo Clinic College of Medicine, Rochester, Minnesota, United States; d The Maastricht Multimodal Molecular Imaging Institute (M4I), Division of Imaging Mass Spectrometry, Maastricht University, Maastricht, the Netherlands; e Life and Health Sciences Research Institute (ICVS), ICVS/3B’s, School of Medicine, University of Minho, Braga, Portugal

**Keywords:** Sphingolipids, Neurodegeneration, Neuroinflammation, Psychiatric diseases, Lipidomics, Biomarkers, Surrogate markers

## Abstract

Lipids play an important role in neurodegeneration, neuroinflammation, and psychiatric disorders and an imbalance in sphingolipid levels is associated with disease. Although early diagnosis and intervention of these disorders would clearly have favorable long-term outcomes, no diagnostic tests currently exist that can accurately identify people at risk. Reliable prognostic biomarkers that are easily accessible would be beneficial to determine therapy and treatment response in clinical trials. Recent advances in lipidomic investigation methods have greatly progressed the knowledge of sphingolipids in neurodegenerative and psychiatric disorders over the past decades although more longitudinal studies are needed to understand its exact role in these disorders to be used as potential tools in the clinic.

In this review, we give an overview of the current knowledge of sphingolipids in neurodegenerative and psychiatric disorders and explore recent advances in investigation methods. Finally, the potential of sphingolipid metabolism products and signaling molecules as potential biomarkers for diagnosis, prognostic, or surrogate markers of treatment response is discussed.

## 1. Introduction

Sphingolipids are a greatly conserved category of lipids, which were discovered in the brain by Johann Ludwig Wilhelm Thudichum more than a century ago [1]. It is therefore not surprising that their denomination as cerebrosides, sphingomyelins, and gangliosides is derived from the initial sources used for their isolation. The common structural characteristic of this diverse and ubiquitous lipid family is a long chain (sphingoid) base backbone [2]. Sphingolipids are important components of lipid bilayers and contribute uniquely to structural and functional properties of the membrane [3]. Together with cholesterol, they are most important elements of detergent-resistant membrane microdomains, also known as lipid rafts [4,5]. Dynamics of the membrane, including vesicular trafficking, are closely related to sphingolipid interconversions [6].

**Fig. 1.**
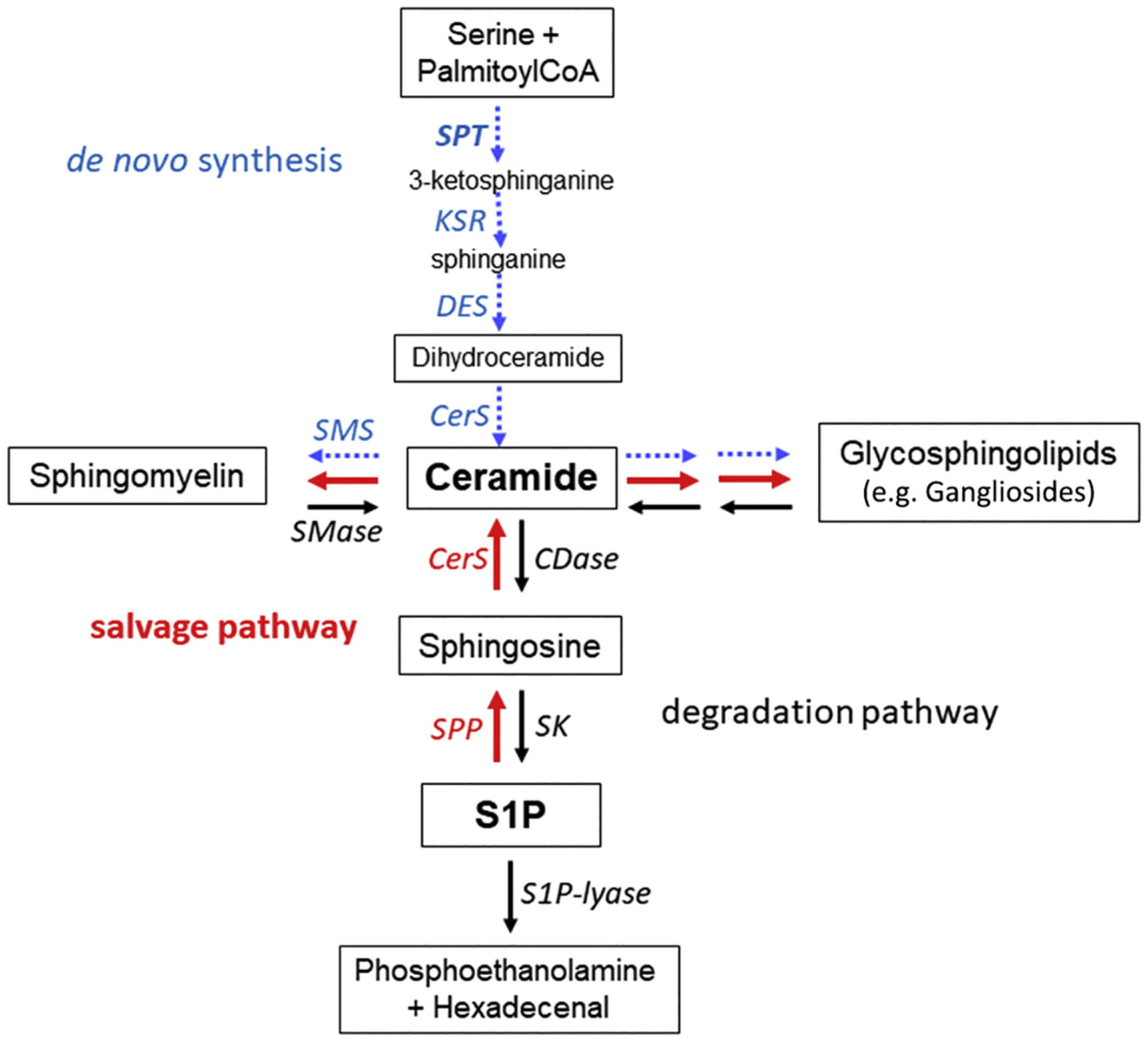
Basic scheme of sphingolipid metabolism. *De novo* generation of ceramide starts from serine and palmitoylCoA (blue arrows) or through recycling of sphingosine 1-phosphate (S1P) (red arrows). Ceramide is the degradation product, as well as the biosynthetic precursor, of all glycosphingolipids and sphingomyelin. Hydrolytic degradation of ceramide (black arrows) generates a fatty acid (not shown) and sphingosine which can, in turn, be phosphorylated to generate S1P. S1P-lyase irreversibly cleaves S1P into phosphoethanolamine and hexadecenal. Abbreviations used are: CerS, ceramide synthases (CerS1 is widespread in the brain); CDase, ceramidase (acid ceramidase is a major glycoprotein in the brain); DES, dihydroceramide desaturase; KSR, ketosphinganine reductase; SK, sphingosine kinases (SK1 and SK2 isoforms are known); SPP, S1P phosphatases (SPP1 and SPP2 isoforms are known); SMase, sphingomyelinases (acid and neutral forms are known); SMS, sphingomyelin synthase; See text for further explanations.

Ceramide is the central molecule in the metabolism of all sphingolipids (Fig. 1). Its formation starts with the condensation of two common cellular metabolites, serine and palmitoyl-CoA, in the *endoplasmic reticulum* (ER). The enzyme catalyzing this rate-limiting step is serine palmitoyltransferase (SPT) [7]. The reaction product 3-ketosphinganine is then produced to dihydrosphingosine (also known as sphinganine). N-acylation of dihydrosphingosine is catalyzed by a family of 6 different ceramide synthases (CerS), each of which uses a restricted subset of acyl-CoAs [8] to generate the respective dihydroceramide. The final step of *de novo* ceramide formation is introduction of a double bond into the sphingoid base moiety by dihydroceramide desaturase (DES) [9]. Ceramide is then either glycosylated in a stepwise manner leading to the generation of glycosphingolipids (GSLs), including the sialic acid-containing gangliosides and lactosylceramide (LacCer), or gains a phosphocholine head group to form sphingomyelin (Fig. 1). Alternatively, phosphorylation of ceramide can produce ceramide 1-phosphate and further continue to synthesize more complex sphingolipids such as, GSLs and sphingomyelin. Interestingly, the transport of ceramide from the ER to the Golgi apparatus where diverse head groups are added, is rather specific: it reaches the site of glycosylation via vesicular transport by the ceramide transfer protein (CERT) which delivers it to the site of sphingomyelin formation [10].

The degradation of GSLs to ceramide primarily takes place in the lysosomes by the action of specific acid exohydrolases, which start from the hydrophilic group [11]. Small glycoprotein cofactors (sphingolipid activator proteins; SAPs) are needed to assist lysosomal exohydrolases to degrade GSLs with three or fewer monosaccharide residues [12]. Insufficient degrading hydrolases and/or SAPs causes the respective substrate in the lysosome to accumulate. Since neurons are especially sensitive to the accumulation of lipids, lysosomal lipid storage disorders (LSDs) like sphingolipidoses or gangliosidosis, often affect the brain [13]. Moreover, complex gangliosides are abundant in the brain and particularly in neuronal membranes [14,15]. Gangliosides are involved in neuronal plasticity and repair, and especially the subtype GM1 has been implicated neurotrophic mechanisms [16]. The degradation of sphingomyelin, and thus recycling of ceramide, is catalyzed by various sphingomyelinases [17]. Since the discovery that membrane sphingolipids could serve in signal transduction pathways and sphingomyelin and ceramide were discovered as a bioactive signaling molecule [18,19], interest in sphingomyelinases and other enzymes and sphingolipids in this pathway has continuously increased, especially due to their function in central nervous system (CNS) physiology [20–24].

Degradation of ceramide starts with its hydrolytic de-acylation to sphingosine, catalyzed either by a ceramidase (CDase) [25] or by diverse non-lysosomal neutral CDases [26,27]. Sphingosine is then phosphorylated by two different kinases (SK1 and SK2) [28], generating sphingosine 1-phosphate (S1P). The two isoenzymes differ by their substrate specificity and subcellular localization. Cytosolic SK1 becomes activated by plasma membrane recruitment and is highly specific for sphingosine. The membrane-associated SK2 is less specific and constitutively active in the ER/nuclear compartment. Note that S1P is not only a sphingolipid degradation product but also an evolutionarily conserved bioactive lipid [29]. The final enzyme in sphingolipid degradation is S1P-lyase (Fig. 1). It catalyzes the irreversible cleavage of S1P to phosphoethanolamine and a long-chain aldehyde [30]. Alternatively, S1P is dephosphorylated by S1P-phosphatases (SPPs; two isoenzymes are known so far) [31]. This reaction is considered as the starting point for the sphingolipid salvage pathway [32] (Fig. 1, red arrows), which was shown to be especially important in terminally differentiated cells, such as neurons, to reestablish complex GSLs [33]. Note that S1P-lyase as well as SPPs are located in the ER.

Sphingolipids are highly abundant in the brain [34] where they form cell type-specific profiles [14] that characteristically change during development and aging [35], and with brain pathological alterations. It was initially shown that metabolic modifications of gangliosides are closely connected to neurodegeneration [15,36–39]. However, during the last decades, the bioactive metabolic intermediates of gangliosides and sphingomyelin, including primarily ceramide and S1P, emerged as critical players in the maintenance of brain health [24,40–43]. For example, an inborn mutated S1P-lyase was recently associated with brain malformations [44]. Other mutations affecting the same enzyme also result in neurological degeneration in addition to several other abnormalities [45]. Importantly, not only the functionality of an enzyme but also the subcellular site where a bioactive lipid is generated appears to affect its signaling function [46]. In line with this assumption, mislocalization of SK2 has been reported in neurodegenerative diseases [47]. Additionally, the CERT protein has also been implicated in neuroinflammation and neurodegeneration by binding to serum amyloid P-component (SAP) and colocalizes in amyloid plaques in the brain of Alzheimer’s disease (AD) patients [48–51].

Taken together, the role of sphingolipid metabolism and related enzymes in neurological diseases are being increasingly investigated. Additionally, modern research techniques allow higher specificity in measuring sphingolipids in the brain, blood, and CSF. This potentially opens doors towards the development of biomarkers indicating specific neurological diseases that can be used in the clinic to improve treatment options.

### 1.1. Lipid investigation methods

Lipidomics as a field has heavily relied upon the high sensitivity of mass spectrometry-based techniques. Recent advances in mass-spectrometry lipidomics now make the detailed lipid composition characterization of a given biological sample possible, which includes different sphingolipid classes and other lipid categories [52]. Initially, experiments were mainly so-called “shotgun” methods, where lipids were extracted from pulverized tissue and injected into the mass spectrometer without further purification or separation [53]. With the advent of electrospray ionization, these “crude extract” methods were useful in determining glycerophospholipids in cellular membranes [54], bile acids [55], and enzymatic substrates [56]. Further evolution of these techniques incorporated liquid chromatography (LC), improving detection by removing ion suppression, as well as enhancing identification by adding a new criterion [57]. Modifications to the general process in LC-mass spectrometry lipidomics have included normal-phase (non-polar solvent) LC for glycerophospholipid (GPL) headgroup identification [58], reverse-phase (polar solvent) LC for acyl chain separation [59], and has been employed to study a variety of neurodegenerative diseases [60–62]. In this context, lipidomics has played a small, but growing role over the past 2 decades. While these techniques have greatly increased the understanding of lipidomics in both plasma and whole tissue, they, unfortunately, fail to separate the fine-scale phenomenon. Probing the spatial distribution of lipids in tissue lies fully within the wheelhouse of mass spectrometry imaging (MSI). By using the most common MSI technique, matrix-assisted laser desorption/ionization (MALDI), several investigators have made strides in identifying sphingolipids that are tied to neurological dysfunction. This is due to a surge in the analytical methods over the past decade, which have allowed higher sensitivity, greater specificity, and finer resolution than ever before [63–65]. These techniques have been extended to examine lipid biochemistry changes in healthy tissues and have been fundamental for lipidomic studies in neurodegenerative diseases. In this review, current knowledge of sphingolipid alterations in neurodegeneration (AD, Parkinson’s disease: PD, and Huntington’s disease; HD), neuroinflammation (multiple sclerosis; MS), and psychiatric disorders is discussed. Additionally, current research models and tools to evaluate lipids in these disorders and the potential of sphingolipid metabolism biomarkers in the clinic is evaluated.

## 2. Sphingolipids in neurodegenerative diseases

### 2.1. Alzheimer’s disease

AD is a neurodegenerative disease resulting in a decline in memory and cognitive disability [66]. The major neuropathological hallmarks of AD are the intercellular accumulation of amyloid (Aβ42) plaques and the formation of extracellular neurofibrillary tangles of hyper-phosphorylated tau protein, which result in impairment of synaptic function and ultimately neuronal death [66,67]. In the past decades, various studies have found that abnormal sphingolipid metabolism is associated with AD pathology. More specifically, increased *de novo* synthesis of ceramide accelerates the production of Aβ in neurons and higher levels of ceramide in the brain are seen in AD and other neurodegenerative disorders [68,69]. Additionally, ceramide species are elevated in CSF and brain white matter, which especially peaks in very mild forms of AD dementia [70,71]. Furthermore, multiple clinical studies suggest a relationship between plasma ceramides and dementia in AD, linking high levels with memory decline among individuals with mild cognitive impairment (MCI) and AD [72–74]. Other studies reported that high plasma ceramides were related to hippocampal volume loss [75–77]. Since these studies additionally suggest that brain and plasma ceramide levels are altered at an early stage of AD, research focusing on the metabolism of ceramide as a target for the development of blood-based biomarkers has been ongoing, although further studies are necessary to make definitive conclusions about the potential of blood ceramides as an indicative tool for AD.

In addition to ceramide, other potential targets in sphingolipid metabolism (e.g. sphingomyelin, sphingosine, sphingomyelinase, and SK1) have also been found (Table 1) [78–80]. Higher levels of sphingomyelin inhibit the activity of γ-secretase in the APP cleaving process, thereby reducing Aβ accumulation [81]. However, in AD, Aβ promotes sphingomyelinase activity leading to ceramide increase in the brain. Ceramide, in turn, stabilized BACE-1 which helps cleave the APP protein to form Aβ peptides in AD [78,80]. Although ceramide can be converted into sphingosine, an increase in sphingosine is also seen in the AD brain. Since sphingosine can be phosphorylated to form S1P, which is reduced in AD [80], this pathway may be of interest when researching pharmacological targets for the disease. FTY720 (fingolimod) is an S1P receptor agonist and approved treatment for MS which has shown to modulate neuroinflammatory pathways in an AD mouse model [82,83]. Since almost all neural cells express S1P receptors [84], fingolimod is considered an interesting target of investigation as a potential AD drug treatment.

Besides ceramides and the S1P pathway, alterations of more complex GSLs have been observed in AD [38]. Six percent of the total amount of lipids in the brain consists of gangliosides [85]. In AD, GM1 levels are elevated and seem to be particularly associated with Aβ-plaques [38]. More specifically, toxic accumulation and aggregation of Aβ is seen when ganglioside GM1 is enriched in lipid rafts [38,86]. This observation indicates a strong connection between ganglioside homeostasis and AD. Kaya *et al.* probed the lipid microenvironment of Aβ plaques, discovering localization of several species of gangliosides and ceramides (specifically GM2, GM3, Cer(d18:1/12:0), and Cer (d18:1/14:0)) [87]. These microenvironments correlate with the largest lipidomics changes throughout the hippocampus and the cortex in AD. Similarly, Sugiura *et al.* studied the accumulation of gangliosides in developing mouse brain [88]. Gangliosides are typically formed with a long-chain sphingoid base (LCB) of 18 or 20 carbons, with C18 gangliosides being found throughout the nervous system, and C20 gangliosides being localized purely within the CNS [89–91]. However, even within the context of the brain, C20 gangliosides accumulate only within the entorhinal-hippocampus projection during development [88]. As the brains aged, C20 gangliosides in the form of GM1 (GM1(d20:1/C18:0) accumulated in the outer molecular layer of the dentate gyrus where neuron degradation occurred similar to AD progression [92].

Together, these results suggest that ceramides, sphingomyelin, S1P, as well as more complex GSL, are associated with AD development and progression although further research is needed to clarify the underlying mechanisms. Animal models of AD might be a useful tool to investigate lipid metabolism in AD. In a lipidomic analysis where human late-onset AD brain tissue was compared to transgenic familial-AD (FAD) mouse-models with mutations in presenilin 1 (PS1) and APP genes, the authors show that, although many lipid alterations were different between the two models, ganglioside (GM3) and cholesterol esters (CE; components of circulating lipoproteins), in particular, showed that disease-related alterations in brain concentration were comparable between the human and animal model [93]. Another study, showed elevated GM3 levels in the human frontal and parietal cortex [94]. Although differences in AD etiology between the models should be considered and may largely explain variation in lipid alterations, these results indicate that FAD mouse models of AD are useful tools for studying aberrations in lipid metabolism in AD and proof to be particularly relevant models in translational research studies.

### 2.2. Parkinson’s disease

PD is the most common progressive neurodegenerative disease after AD [95]. Symptoms include tremor, bradykinesia, rigidity, and loss of postural reflexes together with behavioral disorders and cognitive decline [96]. Symptoms of PD result from cell loss in the substantia nigra and cognitive and behavioral symptoms among PD patients typically result from over-aggregation of Lewy bodies containing α-synuclein inducing dopaminergic neurons damage [95,97,98]. PD, in contrast to AD, is relatively new to scrutiny within lipidomics. The main works have focused on α-synuclein as the principal agent in PD [99], but more recent studies have examined the downstream effects of α-synuclein toxicity. These have found that α-synuclein impacts lipid homeostasis, leading to PD observed neurotoxicity [100].

Nevertheless, it has been indicated that sphingolipids play an important part in PD development [101–103]. Glucocerebrosidase (GBA, the enzyme breaking down GlcCer into glucose and ceramide) has been implicated in sporadic PD and Lewy Body Dementia since mutations in the gene coding for this enzyme is one of the most established genetic risk factors for these diseases [104]. Additionally, ceramide and sphingomyelin show an abnormal metabolism in PD postmortem brain tissue [101] and in the plasma of PD patients with cognitive impairment, various ceramide species (C16:0, C18:0, C22:0, C24:1) were found to be increased [102]. However, in particular, a loss of GM1 gangliosides is seen in the disease [103]. Consequently, animal models in which GM1 levels are reduced develop PD-like motor symptoms. More traditional models in which α-synuclein overexpression is induced (A53T) or dopaminergic neurons are damaged by administration of 1-methyl-4-phenyl-1,2,3,6-tetrahydropyridine (MPTP), have shown SK2 and S1P to promote dopaminergic neuronal survival [105]. Given the effect of fingolimod on S1P receptors, this drug could be a potential modulator for PD patients too. Interestingly, in an A53T transgenic mouse model, fingolimod reduced α-synuclein aggregation and increased brain-derived neurotrophic factor (BDNF) levels in aging mice [106]. More recently, in a PD mouse-model using specific GM2+/− transgenic mice to reduce GM1 gangliosides, the additional beneficial effect of fingolimod administration on motor-behavior was shown [107]. The authors argued that, since normal GM1 levels help to solubilize α-synuclein and that the decreased ganglioside levels in PD could be restored by GM1 treatment, a GM1-deficit mouse model with subsequent motor deficits would make a good animal model for PD. In this model, fingolimod improved movement, sensorimotor function, and urinary tract function in aging GM2+/− mice. Although these effects of fingolimod are promising, other animal studies that used MPTP administration to induce PD-like symptoms did not show neuroprotective effects of fingolimod administration but found that the increase of BDNF by fingolimod was unsustainable over time after multiple injections [108]. Since the known studies using fingolimod in PD have relied heavily on animal research, clinical studies might give valuable insight to its potential beneficial effects in PD patients. Additionally, in a five-year open study, long-term administration of GM1 has been proven safe in humans and clinical benefits for PD patients have been observed [109]. In this controlled randomized double-blind placebo study, PD patients who had received five years of GM1 treatment, reported lower Unified Parkinson’s Disease Rating Scale (UPDRS) movement ability and Activity of Daily Living grades [109]. However, treatment effects currently remain symptomatic and fundamental research on the pathogenesis of PD and the role of sphingolipids is needed.

### 2.3. Huntington’s disease

HD is an inherited progressive neurodegenerative disorder in which disturbances are seen in motor, psychiatric, and cognitive function that ultimately leads to pneumonia, heart damage and lifespan decline [110–112]. Abnormal expansion of a CAG triplet repeat in the Huntingtin (HTT) gene is causative for HD. The HTT gene has been recognized to play various roles in intracellular functions including protein trafficking, regulating transcription and post-synaptic signaling, as well as proteotoxicity and protein aggregation [113–115]. Although not as abundant as in AD and PD, over the recent years, various lines of research have been focusing on the sphingolipid metabolism in HD, in which gangliosides and the S1P pathway seem to be primarily involved in the disease [116–123].

In 2017, Di Pardo, et al. showed a significantly altered expression of S1P-metabolizing enzymes in HD. More specifically, S1P Lyase1 (SGPL1) was found to be upregulated and sphingosine kinase 1 (SK1) downregulated in post-mortem striatum and cortex of patients with advanced HD compared to control [118]. Additionally, in an HD transgenic mouse model (R6/2) expressing exon 1 of the human HTT gene carrying 141–157 CAG repeats [124], an overexpression of ceramide species C20:0, C22:0, C24:0, and C24:1 was seen in the striatum, while ceramide C24:0 was found to be upregulated in the cortex of these mice [118]. Conversely, the expression of S1P was decreased. Interestingly, the authors were additionally able to show that ceramide and SGPL1 levels were already increased in an early stage of the disease (6 weeks old mice), while no differences in SK1 and 2 expression was seen. These results suggest that S1P metabolism is affected in HD. Consequently, stimulating S1PRs via fingolimod has been studied as a therapeutic strategy for HD [125]. In 2014, Di Pardo, et al. showed that fingolimod improved motor function and coordination in the open-field test and horizontal ladder task, and activated pro-survival pathways AKT and ERK in R6/2 mice striatum and seemed to reduced brain atrophy [119]. Furthermore, in the same study, fingolimod was able to reduce mutant huntingtin aggregates. More recently, indirect promotion of S1P production by stimulation of SK1 through the hydrophobic “pseudo-ceramide” compound K6PC-5 [126], showed similar favorable effects in R6/2 mice [121]. Importantly, while GM1 levels were decreased in R6/2 mice striatal areas, fingolimod administration restored GM1 expression to non-pathogenic levels [119].

Previous research has also indicated an impaired ganglioside metabolism in HD [116,123]. A reduction of ganglioside GM1 was found in fibroblasts from patients with HD as well as in HD animal models [123]. In turn, this GM1 reduction leads to HD cells that are more prone to apoptosis. Additionally, in a yeast artificial chromosome (YAC) transgenic HD mouse-model, where the whole human mutant huntingtin protein with 128 CAG repeats is expressed (YAC128) [127], administration of GM1 resulted in lower levels of the mutant huntingtin protein [116]. Furthermore, various other beneficial effects, such as improved dopamine and serotonin metabolism modulation and glutamate, GABA, L-Ser, and D-Ser level restore were also observed [116]. Moreover, the same study showed that GM1 treatment in R6/2 mice decelerated neurodegeneration, white matter atrophy, and loss of body weight. While no true therapeutic measures are yet available for HD, recent studies have shown that the subventricular zone in the human brain is a region of interest, due to the wholesale loss of lipid architecture within the 4 laminae in HD [128].

### 2.4. Biomarkers for neurodegenerative diseases

Taken together, these results show that sphingolipid metabolism has an important function in neurodegenerative diseases, although the exact role of sphingolipids in these disorders remains elusive. Results from previous studies indicate that GM1 treatment and modulation of the S1P pathway may be interesting targets to pursue in future clinical trials in neurodegenerative diseases. However, concerns about the poor blood-brain barrier (BBB) permeability of GM1 [88] and the limited understanding of the therapeutic effects of S1P-pathway modifying drugs in animal models should be considered and further pre-clinical research is necessary before modulating the sphingolipid metabolism in future clinical trials. Additionally, although gross disruptions in sphingolipid metabolism are seen in these neurological disorders, recent studies propose that several neurodegenerative diseases are contributed by more subtle changes in sphingolipid balance. Indeed, plasma ceramide levels have been shown to be altered in MCI and AD dementia [72,74,134–137] and plasma ceramide, GlcCer, and ganglioside levels were altered in PD or Lewy Body dementia patients [102,138,139]. Thus, the utility and context of use of plasma sphingolipids for clinical purposes including diagnosis, prognosis, and therapeutic aspects is warranted. Additionally, sphingolipids are being investigated for their potential as surrogate markers of treatment response or to predict who is most likely to respond. For example, a Phase 2 clinical trial of ibiglustat for PD GBA mutation carriers is ongoing. Ibiglustat is proved to be a small-molecule inhibitor of GlcCer synthesis. If proven successful, the measurement of GlcCer could be clinically useful in the future for determining ad-equate response to therapy and drug dosage assesment.

## 3. Multiple sclerosis

MS is an inflammatory disease, associated to autoimmunity that is characterized by demyelination of the CNS [140,141]. It is estimated to affect one in 1000 North American and European inhabitants, with a markedly greater prevalence in women [142]. Generally, three stages can be defined in the disease. First, in the preclinical stage, the disease is triggered by various genetic and environmental factors yet symptoms remain below the clinical threshold. In the second, relapsing-remitting (RRMS) stage, clinical manifestations start to emerge, characterized by episodic neurologic dysfunction and remissions. Disabilities and deterioration greatly increase in the third, progressive clinical stage, in which sensory functions and gait are particularly affected [140]. Since disease-modifying drugs that target the progressive form of the disease remain largely ineffective, additional research on its fundamental pathogenic mechanism is needed [140,143].

Several lines of research have suggested a role for sphingolipids in MS [24,143–147]. Particularly, the diverse role of ceramides, sphingosine, and S1P are increasingly researched in relation to the process of myelination [24,144,145]. While different ceramide species are primarily involved in neurodegenerative diseases, such as AD, sphingosine in particular seems to be associated with MS. Specifically, sphingosine accumulations in the MS brain have been reported [24]. It is suggested that these sphingolipids influence the course of myelination during early brain development. However, in the diseased state of MS, ceramide and sphingosine are involved in CNS demyelinating processes [24]. Conversely, S1P receptors may be important in decreasing demyelination and, at the same time, improving the course of re-myelination in MS [145]. More specifically, it has recently been shown that inactivation of the S1P receptor 2 (S1PR2) reduced demyelination and lowered clinical disability scores after experimental autoimmune encephalitis (EAE) was induced in mice. In general, a hyperactivation of the sphingomyelin-ceramide-sphingosine-S1P pathway has been suggested to be an underlying mechanism resulting in an increased total phospholipid and decreased sphingolipid levels in normal appearing white- and grey matter in MS [147].

One of the key hallmarks of MS is a progressive loss of oligodendrocytes and, consequently, the degradation of neuronal myelin sheets [148]. Since sphingolipids are particularly abundant in myelin sheets, current research efforts focus on investigating the importance of sphingolipids in demyelination. The hyperactive sphingomyelin-ceramide-sphingosine-S1P pathway seen in MS may lead to dysregulation of the delicate sphingolipid balance in CNS white - and grey matter [147]. Following this line of investigation, recent research suggests a sphingosine-induced toxicity in oligodendrocytes [24,144]. As a case in point, in an experiment to determine cellular toxicity of sphingosine, a significant increase in human oligodendroglioma (HOG) cell death was observed after a high dose of sphingosine was administered to these cells. Interestingly, however, treatment with a low concentration of sphingosine seemed to induce mild cell growth in HOG cells [24]. Research on the mechanism underlying sphingosine toxicity in MS showed an activation of the de novo sphingolipid biosynthesis. Additionally, inhibiting this pathway by myriocin blocked ceramide elevation and protected against oligodendrocyte apoptosis [144].

### 3.1. Sphingosine-1-phosphate receptors in multiple sclerosis

**Fig. 2.**
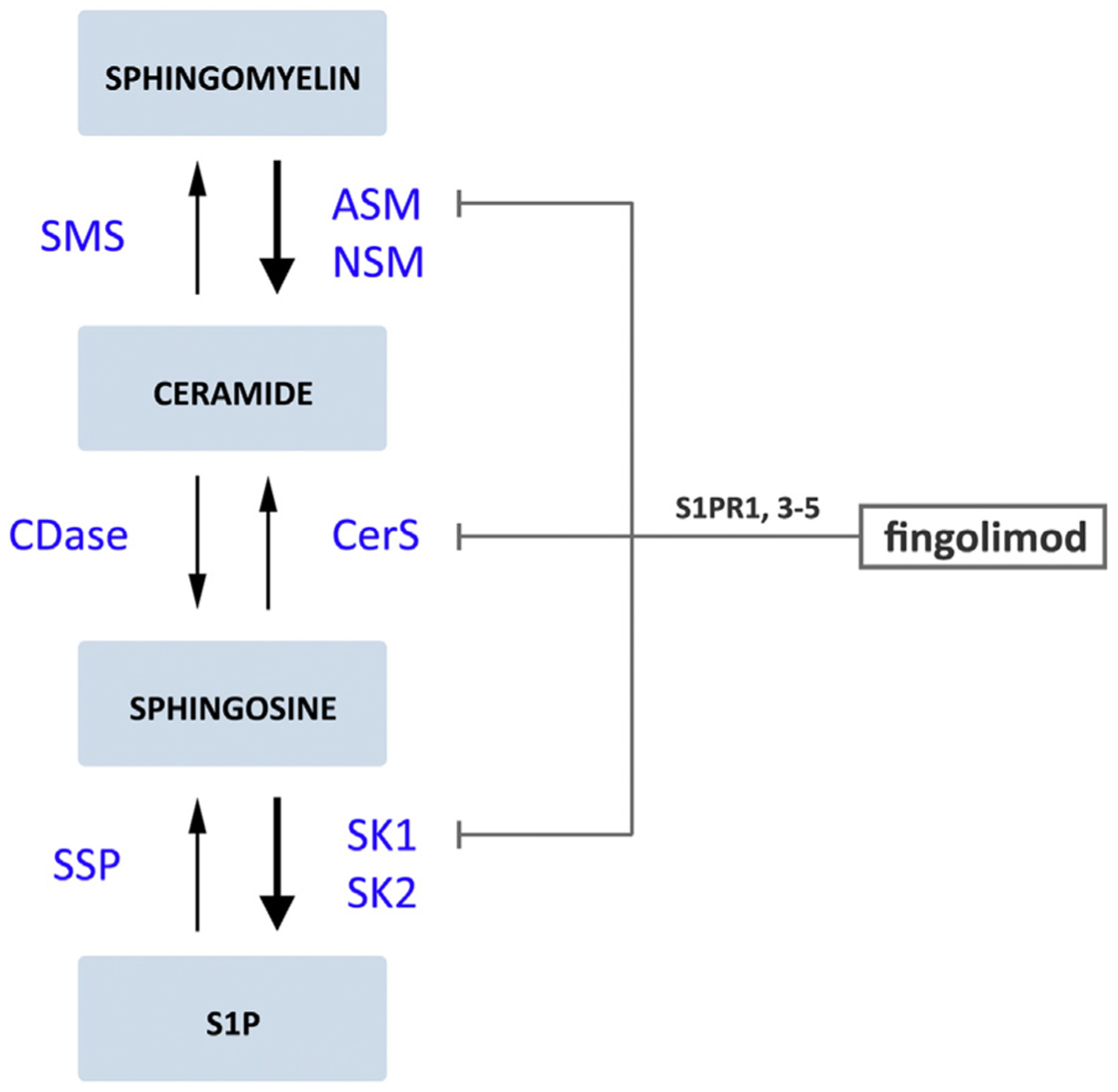
The effects of FTY720 (fingolimod) on a hyperactive sphingomyelin-ceramide-sphingosine-S1P pathway in MS. The thick arrows indicate the hyperactive part of the pathway in multiple sclerosis (MS) [146,147]. Abbreviations used are: S1P, sphingosine-1-phosphate; S1PR, S1P receptor; SMS, sphingomyelin synthase; ASM, acid sphingomyelinase; NSM, neutral sphingomyelinase; CDase, ceramidase; CerS, ceramide synthases; SPP, S1P phosphatases; SK, sphingosine kinases (SK1 and SK2).

SK1 and SK2 phosphorylate sphingosine to form S1P. Besides the ability to influence cellular properties such as cell-survival, S1P is particularly implicated in regulating the immune system by the ability to bind to S1PR1–5 [149]. Taking into account the role of inflammation in MS development, S1PRs have been interesting targets of pharmacological potential in MS [150]. Fingolimod is a drug that modulates S1PR1, 3–5 and is able to cross the BBB [143,151]. Consequently, it was the first FDA approved oral drug for the relapsing form of MS, entering the market in 2010 [83]. There is evidence that fingolimod works by targeting astrocytes that give rise to increased levels of acid sphingomyelinase (ASM) in MS brain lesions where it has an anti-inflammatory effect [146,152]. Since ASM converts sphingomyelin into ceramide, this leads to an upregulation of ceramide production in the MS brain. It was shown that primary astrocytes that were exposed to tumor necrosis factor-α (TNF-α), a pro-inflammatory cytokine, enhanced ceramide production by increasing the expression of ASM [146]. Interestingly, subsequent treatment of fingolimod on these primary astrocytes reduced this effect. Additionally, fingolimod inhibited SK1 [153] and ceramide production via CerS inhibition [154,155] (Fig. 2). Furthermore, recent evidence from an EAE secondary progressive MS mouse model suggests that fingolimod decreases neurodegeneration and neurotoxicity by modulating astrocyte activation via S1PRs [143]. Specifically, fingolimod binds to the S1PR1, causing the receptor to internalize and thereby lowering the response of lymphocytes to emigrate from lymph nodes [146].

Fingolimod has been established as a safe and effective drug with great benefits for a broad range of patients with MS. However, its side effects due to nonspecific targeting of S1PRs have to be considered. The FDA recently contraindicated fingolimod for patients with preexisting heart conditions or previous stroke episodes [151,156]. Although various studies have investigated the role of S1PR1 in MS, S1PR2–5 have received considerably less attention in MS research. However, some recent studies discuss these receptor isotypes and their pharmacological potential in more detail [145,157–162]. In 2018, Seyedsadr et al. described the role of the S1PR2 [145]. In this study, the authors show that activation of the S1PR2 has a demyelinating effect in MS and, additionally, impairs myelin repair. Moreover, knock-out of the S1PR2 gene, as well as administration of the pharmacological S1PR2 inhibitor JTE-013, reverses these damaging effects. Furthermore, in another study, S1PR2 has been shown to have sex- and strain-specific effects on BBB permeability regulation [158]. Studies that describe S1PR3 and S1PR4 in relation to MS remain limited. However, it has been suggested that the action of fingolimod to protect astrocytes against neuroinflammation depends in part on S1PR3 activity [159]. S1PR4, together with S1PR1 and S1PR2, are believed to be involved in lymphatic CD4 T cell migration [162] and selective S1PR4 agonists and inhibitors with no activity against the other S1PRs are currently emerging [160]. S1PR5 is particularly expressed in the CNS and is involved in early inflammatory processes [163,164]. A more promising novel S1PR-modulator for MS treatment might be BAF312 (siponimod) which selectively targets S1PR1 and S1PR5 [157]. Importantly, siponimod, in contrast to fingolimod, does not require to be phosphorylated to be effective and exhibits no activity against S1PR3 and S1PR4 [165]. After a successful phase 3 trial, termed the “EXPAND” study, where siponimod was tested against placebo in secondary progressive MS [161], FDA approval was given in 2019, making it the first oral drug treatment for secondary progressive MS with active disease [166].

### 3.2. Biomarkers for multiple sclerosis

Although various lines of research give further insight into the mechanism driving MS, early diagnosis and subsequent early treatment remains difficult and methods to identify and observe immunological and neurodegenerative processes in MS pathology are lacking [167,168]. With recent advances in lipidomics research methods, the interest in lipids as biomarkers for MS is growing. Interestingly, although sphingosine and S1P are believed to be crucial molecules in the pathogenesis of MS, few reports have indicated these sphingolipids as potential targets for MS biomarkers.

In 2011, an LC mass spectrometry lipidomic analysis was performed on MS patient serum and compared to healthy controls and patients with other neurological diseases (OND) [169]. Although sphingolipids like galactocerebroside (GalCer, a neutral glycosphingolipid) and sphingomyelin were also measured, the metabolism of phospholipids (the main component of cell membranes), in particular, was altered in the serum of MS patients. Specifically, an altered ratio of glycerophosphatidylcholine (PC) and lyso-PC levels was found [169]. An evaluation of S1P concentrations in the blood and CSF of MS patients revealed that S1P levels in the blood did not differ from the control group, but CSF levels were significantly higher in MS patients [170]. Further analyses demonstrated the toxic effect of CSF from MS patients to neurons [171]. Rat neurons that were exposed to CSF from MS patients showed oxidative stress and a decreased expression of neuroprotective genes. Additionally, levels of ceramide species C16:0 and C24:0 were found to be specifically increased in the CSF of MS patients, leading to the observed toxicity in neurons. More recently, several sphingomyelin species were found to be reduced in MS CSF compared to OND [172]. Importantly, to understand the mechanism underlying this decrease, ASM expression and activity in CSF was further investigated by an enzymatic activity assay to assess its role in the sphingomyelin pathway in MS. This revealed that low levels of sphingomyelin in the CSF results from overexpression of ASM carried by specific exosomes [172]. Further lipidomics and subsequent receiver operating characteristic (ROC) analyses where MS patients were compared to a subgroup of inflammatory neurological diseases, showed that ASM activity in the CSF might be considered as an interesting biomarker for MS that is able to discriminate disease state, independent of other inflammatory and neurological conditions.

Besides S1P, ceramides, and ASM, other lipid species have also been the target of investigation of potential biomarkers for MS. It has been shown that levels of various sulfatide (sulfated GalCer) species are significantly altered in MS. Specifically, total sulfatide levels were found to be reduced by 60% in human MS plaque tissue compared to control tissue [173] and, in the plasma of RRMS patients, sulfatide C18:0 and C24:1 levels positively correlate with a higher disease disability [174]. Recently, in a non-targeted lipidomic approach investigating 9532 lipid species in the CSF of MS and non-MS patients, 47 species, of which three sphingolipids, were identified based on their mass and were significantly altered in MS (glucosylceramide, GlcCer(D42:0) upregulated; C20 sulfatide and ceramide-phosphate (42:2) downregulated) [175]. Although these results contribute to the understanding and potential development of sphingolipid-related biomarkers in MS, CSF collection remains relatively invasive and studies investigating alternative biomarkers that are more easily accessible are ongoing. Interestingly, a recent report discussing the lipidomic and metabolomic analysis of tears as a possible biomarker in MS showed several lipid species to be altered in the lacrimal fluid in MS patients, including multiple sphingomyelin species [167]. Since five of the thirty potential tear biomarkers corresponded to the ones found in CSF, the authors argue that lacrimal fluid could be considered as an intermediate between serum and CSF.

Taken together, the role of sphingolipids in MS is evident and the SM-ceramide-sphingosine-S1P pathway is an interesting target for disease-modifying treatment options. However, effective therapy remains limited and a need for reliable biomarkers for MS is evident. Although it has been shown that ASM expression is altered in MS, recent advances investigating ASM as a potential blood-based biomarker revealed no correlation with disease activity, progression, or response to treatment [141]. Nevertheless, specific ceramide- and sphingomyelin species require additional investigation. S1PR modulation shows promising potential but further research investigating S1P receptor specificity in MS remains necessary.

## 4. Sphingolipids in psychiatric diseases

**Fig. 3.**
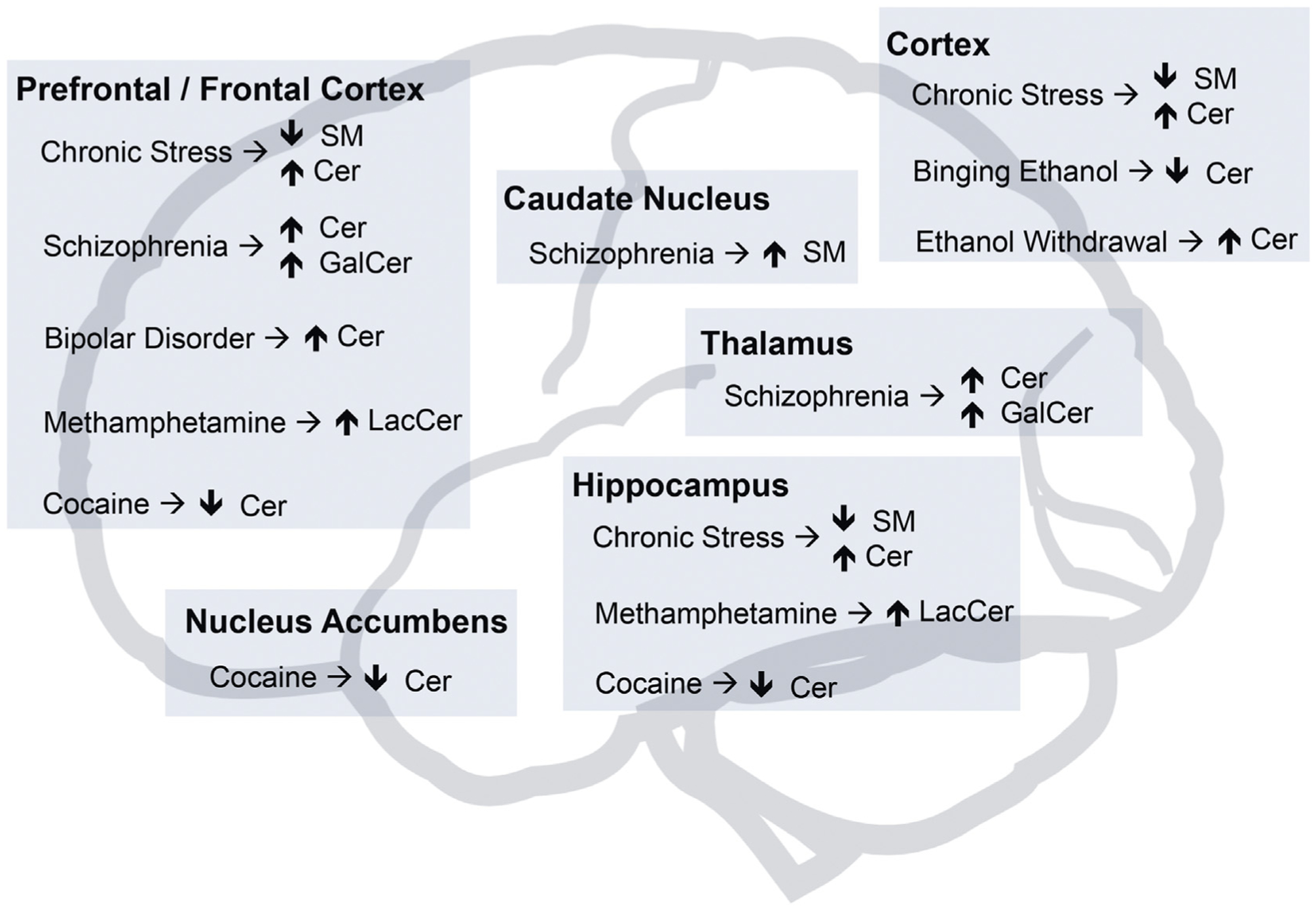
Brain regional sphingolipid metabolism impairment in psychiatric disorders. Summary from various studies of alterations observed in sphingolipid metabolism at the brain regional level in human post-mortem psychiatric patients or rodent models of psychiatric diseases such as exposure to chronic stress or to substances of abuse associated with addiction. SM, sphingomyelin; Cer, ceramide; GalCer, galactosylceramide; LacCer, lactosylceramide.

The brain is a complex organ organized in tightly regulated circuits and the study of neurodegenerative diseases highlighted the concept that selective brain regions are more vulnerable to pathological processes, such as the atrophy of the medial temporal cortex in AD or substantia nigra in PD [176]. Even though neuropsychiatric disorders do not show such a clear anatomic correlate observed by regional atrophy in humans, our current understanding of these disorders shows that the dysfunction of brain-specific regional circuits is at the basis of the characteristic behavioral alterations observed in each disease [177]. Therefore, one hypothesis is that the specific molecular composition of each brain region, namely the lipidomic composition, could be a key contributing factor for either vulnerability or resilience to pathological processes in neuropsychiatric disorders (Fig. 3) [178]. An interesting observation is that patients with mutations in GSL metabolizing enzymes have been indicated to have characteristic clinical psychiatric presentations, as previously reviewed [179]. However, direct extrapolations from patients with enzymatic deficiencies should be done cautiously. Firstly, these mutations usually affect multiple cell types beyond the CNS, and secondly, they impair in many circumstances the endolysosomal flux, which, as a general disease mechanism, is shown to contribute to neurodegeneration [180]. Therefore, studies addressing the role of sphingolipid metabolizing enzymes beyond its role in neurodegeneration should be carefully performed in order to give insight concerning the complex regulation of regional synaptic functioning, brain circuits, and behaviors.

The etiology of neuropsychiatric disorders is multifactorial, with combined genetic and environmental factors, such as the exposure to chronic stress, a known risk factor anxiety and depression development [181]. The use of rodent models that are either exposed to chronic unpredictable stress (CUS) or exogenous administration of corticosterone (one of the biological surrogates of stress exposure), not only show anxious-like behavior, but also present deficits in learning and memory which co-exist with dendritic atrophy in the prefrontal cortex (PFC) and the hippocampus [181]. Importantly, it was shown that chronic stress exposure altered the brain lipidomic composition [178,181]. At the regional level, the PFC was the area showing a higher degree of lipid disturbance and also the hippocampus, to a lesser degree [181], in accordance to previous studies that highlighted the susceptibility of these regions to chronic stress-induced effects [181]. Interestingly, chronic stress was shown to induce ceramide increase and a sphingomyelin decrease in both the PFC and hippocampus, which suggests that sphingomyelinase activity could be increased in stress conditions [181]. Another lipidomic study evaluating the effects of chronic antidepressant drug treatment using maprotiline and paroxetine, showed a decrease in sphingomyelin and various ceramide species increases selectively in the PFC, while no significant effects were observed with fluoxetine [182]. Moreover, it was shown that both CUS and corticosterone exposure leads to increased hippocampal ceramide levels and that ASM, that synthesizes ceramide from sphingomyelin, is a target for the antidepressant drugs, amitriptyline, and fluoxetine [22], further supporting a role for ceramide as a pathological driver of stress effects. Since the PFC presented relatively abundant sphingomyelin levels [181], regional brain lipidome could also partially explain the elevated susceptibility to higher ceramide levels upon chronic stress exposure.

Concerning the hippocampus, recent studies showed that it is differentially regulated by dorsal to ventral sub-regions along its longitudinal axis in the rodent, or from posterior to anterior poles in humans [183]. It is generally understood that the dorsal hippocampus, in particular, is implicated in cognitive functions like spatial memory and that emotionally mediated responses are regulated by the ventral hippocampus [183]. Moreover, the dorsal and ventral hippocampus have different intrinsic electrophysiological properties in response to chronic stress [184,185]. At the level of lipidomic analysis, the rodent dorsal hippocampus shows enrichment in complex GSLs and lower levels of simpler sphingolipids, such as sphingomyelin and dihydrosphingomyelin (dhSM), comparatively to the ventral hippocampus [186]. Additionally, the ventral hippocampus was more sensitive to chronic corticosterone administration at the lipidomic level, showing not only increased levels of cholesteryl esters, triacylglycerol, and phosphatidylserine, but also dhSM [186]. Another study observed a relative increase in ceramide dorsal to ventral hippocampal levels in a relearning extinction paradigm [187], which overall, further supports that the subregional hippocampal lipid composition can be a key contributor to stress susceptibility, potentially affecting circuits predominantly involved in either emotional output or learning and memory [183].

While many advances have been made in the understanding of depression and anxiety through the use of chronic stress animal models, other psychiatric diseases such as schizophrenia, bipolar disorder, and addiction present a bigger challenge. A study on schizophrenic patients identified a gene expression profile in the PFC with increased levels of genes involved in sphingolipid metabolism [188]. Studies on brain lipid composition showed ceramide species levels increased in PFC white matter post-mortem samples of both schizophrenic and bipolar patients [189] and sphingomyelin and GalCers reduced levels in the thalamus of schizophrenic patients [190]. Conversely, other studies on schizophrenic patients showed increased GalCer levels in the frontal cortex [191] and increased sphingomyelin levels in the caudate [192]. Finally, exposure to the bipolar disorder drug valproate leads to increased ceramide in a yeast model [193] and chronic treatment with antipsychotic drug haloperidol altered the normal metabolome affecting significantly sphingolipid related pathways in the mouse brain [194]. Although it is not clear if a single lipid modulating pathway is at the root of the pathophysiology of these psychiatric disorders, overall these observations support a dysregulation of sphingolipid metabolism.

Concerning addiction, while the study of the brain circuits relevant for addictive-like behaviors is fundamental for its understanding and devising new therapies, there are a number of studies that tackle the direct impact of addictive substances exposure on sphingolipid brain metabolism. For instance, chronic ethanol consumption was shown to upregulate ceramide levels and decrease sphingomyelin levels in adult mouse brain [195] and while binging ethanol consumption decreased ceramide levels, ethanol withdrawal increased ceramide levels in the mouse cortex [196]; methamphetamine-sensitized mice had higher degree changes in the hippocampus and PFC lipidome, showing in both, decreased LacCer levels [197]; cocaine conditioned mice have widespread lipidomic alterations in the nucleus accumbens, striatum, PFC and hippocampus, notably showing decreased ceramide levels [198]; and using mass spectrometry imaging, administration of morphine, cocaine, and amphetamine in rodents was shown to lead to increased brain sulfatide levels [198].

Upon the identification of altered lipid signaling pathways in psychiatric brain samples and rodent models in lipidomic studies, the use of genetic models that modulate enzymes involved in sphingolipid metabolism highlight not only the susceptibility to altered behaviors but also validate potential drug targets. As an example of this rationale, since increased ceramide levels was one of the most common metabolic hits, modulation of the enzymes that either synthesize or metabolize ceramide could be tested concerning the impact on relevant behavior or resistance to environmental factors that predispose to neuropsychiatric disorders. In line with that, while ASM overexpression led to depressive-like behavior [22] and increased alcohol consumption [199], ASM heterozygote mice were resistant to chronic stress effects [22] and alcohol consumption associated behaviors [199], which indicates the potential of ASM as a therapeutic target. However, the complex consequences of ceramide modulation are highlighted by various studies that show deleterious effects by both blocking ceramide degradation or synthesis through either ceramidase or CerS, respectively. This is observed upon acid ceramidase deficiency, which leads to compulsive and anxiety-like behaviors, among multiple other behavioral alterations [200]; CerS1 knock-out (KO) mice have decreased levels of both ceramide C18 and gangliosides in general, alterations in neurodevelopment and various behaviors such as impaired locomotion and impaired spatial working memory [201]; CerS6 KO mice, which present decreased ceramide C16 brain levels, have impaired open field exploration and habituation [202]; or carnitine palmitoyltransferase 1C KO mice that have reduced ceramide levels, increased immature spines in the hippocampus and deficits in spatial learning [203]. Therefore, lipid modulation should be targeted taking into account the enzymatic expression on subcellular organelles, specific cells, brain regions, and the impact on specific lipid species and other lipids classes, which could all differentially affect behavioral output.

Interestingly, fingolimod has been also shown to decrease anxiety [204] and improve depressive symptoms in MS treated patients [205]. Studies in mice further supported a protective role for fingolimod upon chronic stress exposure [206] in a neurogenesis dependent way [207], as a facilitator of fear memory extinction [208] and in reversing the anxiety-like behavior induced by the regional expression of α-importin 5 in the ventral hippocampus [209]. These suggest that the effects of fingolimod could go beyond its reported mechanism of action of decreasing lymphocyte recruitment to the CNS and further support for a potential role for sphingolipid modulating drugs in neuropsychiatric disorders.

As with neurodegenerative diseases, sphingolipids could be used in neuropsychiatric disorders as surrogate markers of treatment response. An example is the interaction between sphingomyelinases, anti-depressants, and the clinical course of depression. ASM has been shown to be elevated among a small number of individuals experiencing a major depressive episode [210]. Tricyclic anti-depressants such as amitriptyline and fluoxetine, a selective serotonin reuptake inhibitor, are known to reduce ASM activity and ceramide concentrations and to improve behavior in models of depression induced by stress [22]. Thus, a future possibility would be to measure ASM activity and ceramide levels in the blood in an effort to titrate anti-depressant dose. Although a recent study did not find serum ASM activity was useful for predicting the course of clinical depression over a period of three weeks [211], additional studies with longer follow-up and measuring levels of other sphingolipids are needed.

## 5. Clinical utility of sphingolipids for the diagnosis, prognosis, and treatment of neurodegenerative diseases and neuropsychiatric disorders

As described above, there are several lines of evidence highlighting the potential role of sphingolipid pathways in the etiopathogenesis of both neurodegenerative diseases and neuropsychiatric disorders. Indeed, for normal neuronal function, the right balance of sphingolipids in the brain has to be maintained [102], as demonstrated by several severe neurological disorders resulting from enzyme deficiencies in the sphingolipid pathway. Extreme changes in specific sphingolipids, for example GlcCer for Gaucher’s disease, are caused by severe pathologic mutations in the sphingolipid pathway. In these situations, it is possible that plasma sphingolipid levels could be useful to aid in diagnosis, in addition to enzyme levels. However, it is unlikely that plasma sphingolipids will be useful for diagnosing sporadic neurodegenerative diseases such as AD dementia, PD, or Lewy Body Dementia. Studies comparing plasma sphingolipid levels among patients with these diseases to cognitively normal individuals or those in prodromal stages of disease (e.g., MCI) show significant overlap between clinical diagnoses, even if the difference in sphingolipids by group are statistically significant [75,102,137,139]. As a result, sphingolipids cannot be currently used as diagnostic biomarkers for these diseases because they have low diagnostic accuracy and poor sensitivity and/or specificity. Instead, it is more likely that plasma sphingolipids could be used as prognostic markers. Although there are few longitudinal studies, current results consistently suggest that high levels of elevated ceramides indicate a greater rate of cognitive decline among cognitively unimpaired individuals, patients with MCI, dementia, or those undergoing cardiac rehabilitation [72,134–136,212]. Thus, measuring plasma ceramides could be clinically useful for identifying individuals who will have the fastest cognitive decline, especially for identifying fast progressors for clinical trials.

Lastly, sphingolipids could be used in neurodegenerative diseases and neuropsychiatric disorders as surrogate markers of treatment response. Sphingolipid metabolism and associated enzymes play significant roles in neuronal diseases and specific alterations of the sphingomyelin/ceramide/S1P pathway, together with an impaired ganglioside metabolism, are particularly seen. Advanced lipidomic tools to investigate sphingolipid metabolism in neurodegenerative and psychiatric diseases are emerging. This may aid the understanding of sphingolipid metabolism and the potential development of prognostic biomarkers or surrogate markers of treatment response in these neuronal diseases.

## Figures and Tables

**Table 1:** Brain sphingolipid level changes in neurodegenerative diseases.

	Alzheimer’s disease	Parkinson’s disease	Huntington’s disease
Sphingolipids			
Ceramides	Upregulated [70,130]	Downregulated [101]	Upregulated [118]
Sphingomyelin	Downregulated [80]	Downregulated [101]	Not available
Sphingosine	Upregulated [80]	Not available	Not available
Sphingosine-1-phosphate (S1P)	Downregulated [80]	Downregulated [105]	Downregulated [120]
Ganglioside GM1	Upregulated [94]	Downregulated [103,109]	Downregulated [116,114]
Ganglioside GM3	Upregulated [94]	Not available	Not available
Enzymes			
Sphingosine kinase-1 (SK1)	Downregulated [78]	Downregulated [132]	Downregulated [118]
Sphingosine kinase-2 (SK1)	Downregulated [47]	Downregulated [105]	Unchanged[118]
(Acid) Sphingomyelinase (ASM)	Upregulated [80]	Downregulated [133]	Not available
(Acid) Ceramidase (AC)	Upregulated [80]	Not available	Not available
Ceramide synthase (CerS)	CerS1 Upregulated, CerS2 and CerS6 Downregulated [131,129]	Upregulated [101]	CerS1 Downregulated [120]

Upregulation or downregulation of brain sphingolipids and associated enzymes in Alzheimer’s disease (AD), Parkinson’s disease (PD) and Huntington’s disease (HD) [47, 70, 78, 80, 94, 101, 103, 105, 109, 116, 117, 118, 120, 129, 130, 131, 132, 133].
